# Perceived conflict of interest in health science partnerships

**DOI:** 10.1371/journal.pone.0175643

**Published:** 2017-04-20

**Authors:** John C. Besley, Aaron M. McCright, Nagwan R. Zahry, Kevin C. Elliott, Norbert E. Kaminski, Joseph D. Martin

**Affiliations:** 1 College of Communication Arts and Sciences, Michigan State University, East Lansing, Michigan, United States of America; 2 Lyman Briggs College, Department of Sociology, Environmental Science and Policy Program, Michigan State University, East Lansing, Michigan, United States of America; 3 Lyman Briggs College, Department of Fisheries and Wildlife, Michigan State University, East Lansing, Michigan, United States of America; 4 Department of Philosophy, Michigan State University, East Lansing, Michigan, United States of America; 5 Institute for Integrative Toxicology, Michigan State University, East Lansing, Michigan, United States of America; 6 Consortium for History of Science, Technology and Medicine, Philadelphia, Pennsylvania, United States of America; 7 Centre for History and Philosophy of Science, University of Leeds, Leeds, United Kingdom; Iowa State University, UNITED STATES

## Abstract

University scientists conducting research on topics of potential health concern often want to partner with a range of actors, including government entities, non-governmental organizations, and private enterprises. Such partnerships can provide access to needed resources, including funding. However, those who observe the results of such partnerships may judge those results based on who is involved. This set of studies seeks to assess how people perceive two hypothetical health science research collaborations. In doing so, it also tests the utility of using procedural justice concepts to assess perceptions of research legitimacy as a theoretical way to investigate conflict of interest perceptions. Findings show that including an industry collaborator has clear negative repercussions for how people see a research partnership and that these perceptions shape people’s willingness to see the research as a legitimate source of knowledge. Additional research aimed at further communicating procedures that might mitigate the impact of industry collaboration is suggested.

## Introduction

Industry is an increasingly important but controversial source of funding for scientific research and development (R&D) in the United States. Whereas the federal government funded roughly two-thirds of R&D in the U.S. during the 1960s and 1970s, this relationship is now flipped so that private industry is now funding roughly two-thirds of U.S. R&D [1]. Further, university scientists increasingly are participating in public-private partnerships with industry, government service or regulatory agencies, and/or non-governmental organizations (NGOs) to engage with stakeholders and secure more consistent research funding in the face of budgetary constraints on governmental research funding [2–4].

Nevertheless, public skepticism of industry-funded research (e.g., [5, 6]) might constrain the efficacy and influence of its results. The tobacco industry’s efforts to deny the detrimental public-health effects of its products is a well-known example of activities that have raised public awareness of the possibility that industry-funded science could be less than fully reliable [7]. More recently, publications about the safety testing of industrial chemicals and pharmaceuticals [8–12], as well as about anthropogenic climate change [13–15], may have further contributed to public concerns about the legitimacy of science in which industry has played a role. On the other hand, the simple solution of asking scientific researchers to eschew all industry funding and research collaborations seems unfeasible and undesirable, as a significant amount of important and scientifically sound research would not be performed—to the detriment of society [16].

The question of how researchers might benefit from available industry funding without diminishing the real or perceived quality of their research suggests the potential for a multidisciplinary research program that examines the procedures that might be used to protect research. The current set of studies represents one piece of such a work program and is built on the assumption that efforts to design procedures for protecting research should be informed by information about the types of procedures and/or criteria that generate public confidence in the legitimacy of the resulting research. More generally, the current set of studies seeks to develop an initial understanding of how laypeople’s perceptions of a scientific research collaboration are influenced by concerns about conflicts of interest. As is discussed below, the choice of research partners is conceptualized as a basic way to communicate to others that a research team is concerned about taking multiple viewpoints into account and that no one set of interests will dominate. Other procedures to limit conflict of interest (see the discussion section) are also possible but an initial focus on research partners is meant to help establish a foundation for future work.

Past scholarship suggests that procedural justice concepts from social psychology may offer insights for assessing how laypeople perceive research partnerships, given that such collaborations can be understood as processes that researchers use to control biases and consider alternative viewpoints. After reviewing existing work in this area, we identify selected research questions and derive key hypotheses from procedural justice scholarship. Specifically, we are interested in how different collaborations (i.e., processes) can affect perceptions that research will be done appropriately (i.e., procedural fairness) and the degree to which such perceptions may also shape overall perceptions of the research (i.e., perceived legitimacy). We then discuss our research protocols and present the results from three experiments designed to examine how the inclusion of different partners in a health-related research collaboration influences the perceived procedural fairness and perceived legitimacy of the resulting research. In each experiment, subjects considered a hypothetical research collaboration studying the health effects of transfats in foods (Studies 1 and 3) or the health effects of genetically modified (GM) food (Study 2) that included an agrifood business, a research university, a government agency, and/or an NGO. In our conclusion, we outline a further research agenda for investigating the extent to which certain safeguards designed to reduce conflicts of interest may facilitate the production of high-quality industry-funded research that laypeople perceive as legitimate. In our current set of three studies, we focus on perceptions of conflict of interest and do not make specific claims about the degree to which procedures such as partnership arrangements are actually effective at limiting the effects of conflicted interests.

## Existing scholarship

Given the paucity of theoretically driven work in this area, our project conceptualizes collaboration as a conflict of interest mitigation procedure. It does so to build upon and extend a study that employs well-established procedural justice scholarship (e.g., [17]) to examine perceived conflict of interest in science [18]. Analyzing data from a cross-sectional survey of people who attended Food and Drug Administration (FDA) advisory meetings, McComas, et al. [18] find that attendees’ fairness perceptions were associated both with satisfaction with the committee process and willingness to accept the outcomes from the process (i.e., perceived legitimacy). Our project builds upon that study, as well as the broader procedural justice literature, by using experimental procedures to examine how the inclusion of different partners affects procedural fairness perceptions and, ultimately, willingness to use the research as a legitimate basis for personal or public decision-making.

### Defining conflict of interest

Most scholarship characterizes a conflict of interest as a situation in which an individual or organization has a decision-making role that might allow them to improperly benefit from the decisions they might make. This benefit could be financial, but it also could be personal (i.e., helping a friend) or a combination of the two (i.e., helping a work colleague). A benefit also might include avoidance of harm. Importantly, the existence of a conflict of interest does not require any specific behavior to occur. A conflict of interest is only the potential or tendency for biased behavior to occur (for a longer discussion, see: [19, 20, 21]). A relatively recent U.S. Institute of Medicine (IOM) report similarly defines conflicts of interest as “circumstances that create a risk that professional judgments or actions regarding a primary interest will be unduly influenced by a secondary interest” ([22] p. 6). Primary interests here include issues such as the protection of research integrity, students, and patients, while secondary interests include personal and professional concerns.

Most academic writing about conflicts of interest focuses on issues of disclosure. For example, leading medical journals such as the *Journal of the American Medical Association* ([e.g., [23, 24–26]) and the *New England Journal of Medicine* (e.g., [27, 28]) feature numerous articles reviewing existing disclosure policies and their impacts. Commentaries also suggest potential issues and recommend policy changes (e.g., [29, 30–32]). Such recommendations are indeed a central part of the IOM report cited above [22]. Although disclosure policies are clearly important, they are not a panacea [33]; indeed, some research demonstrates that disclosure policies may decrease the quality of advice that experts provide [34, 35]. Less common are studies on the origins and impact of conflict of interest perceptions.

### Experimental research on conflict of interest

As expected, the scant available scholarship suggests that conflict of interest perceptions have negative impacts on how people view research. A 2010 systematic review identifies 20 relevant peer-reviewed journal articles focused on perceptions of ‘financial ties’ in the context of health and medicine [36]. Most studies find that, when asked directly, research participants report worrying about the impact of such conflicts and wanting to know about such conflicts. More important for our project, the limited experimental research reviewed finds that disclosure (versus non-disclosure) of industry funding decreases perceived research quality [37, 38] and decreases trust in the researchers [39]. More recent research confirms such effects [40]. Further, increasing the relative size of potential financial conflicts decreases trust perceptions [41]. Nevertheless, medical research participants do not appear to be substantially less likely to enroll in clinical trials in the face of conflicts of interest [36, 42].

These earlier experimental studies typically ask subjects to review a research product (e.g., a paper) written by authors either in an industry or an academic position. Our project is novel since we more closely approximate the current reality of research funding and partnerships. Briefly, in our three experiments we randomly assign subjects to one of 15 different combinations of research partners—rather than simply ask subjects to consider a simple study or single research outcome.

### Procedural fairness and the study of conflict of interest

We further bolster the novelty of our project by integrating insights from theory associated with procedural justice scholarship into the study of conflict of interest in science. Research in this area consistently shows that people often perceive decisions as legitimate—even if the person feels the decision may go against their interests—if they believe that such decisions result from fair procedures (i.e., ones that allow stakeholders to have a voice and are free from biases that might arise from conflicts of interest) employed by decision-makers who are seen as interpersonally fair (i.e., actors who are respectful) [43, 44]. Indeed, this focus on the effects of ‘procedural’ rather than ‘distributive’ forms of fairness has emerged from a critique of a rational choice approach to decision-making assuming that people only care about outcomes [45].

The three studies reported below conceptualize research involving potential risks as a ‘decision’ about which an observer (e.g., someone who is trying to decide about whether to consider new evidence) might make legitimacy judgments. The work further views the choice of research partners as a conflict of interest mitigation process that researchers can use to, at least partly, make the decision process more procedurally fair. The logic of considering collaboration as a conflict of interest mitigation process is similar to why one might want to include representatives from different parts of an organization when making decisions about major hiring. Likewise, the logic underlies why a political leader might want to build a coalition that includes different constituencies in a cabinet. The expectation is that having multiple voices participate is likely to limit the effect of any one voice.

The adaption of procedural justice concepts seems important for the current set of studies because past research on conflict of interest does not feature theory prominently (e.g.,[36]). Building on past procedural justice theory can both provide a framework for measurement and analyses in the current work as well as suggest additional areas of future research. As noted above, McComas and her colleagues [18] apply procedural justice theory and find that the degree to which attendees saw FDA advisory committees as following fair procedures was associated with overall satisfaction and willingness to accept committee decisions. However, whereas this earlier study examines perceptions of an existing process, the current work seeks to identify processes that researchers might use to mitigate perceptions of bias.

Although not tested here, two related mechanisms likely underlie the impact of fairness perceptions [46]. The most prominent is that people use procedural fairness as a heuristic to assess an outcome when they are unsure about what would constitute a correct outcome [47, 48]. This argument is consistent with findings that fair processes have little impact on those who see a decision as morally mandated—and thus not uncertain (e.g., [49]). Second, other scholarship posits that fair processes and fair treatment matter because they communicate to those affected by a decision that they are valued members of a social identity group. Research supporting this assertion shows that fair process perceptions have a greater impact within groups than between groups [50, 51]. These underlying mechanisms suggest that a person might use information about the partners in a research collaboration to make heuristic sense of a scientific study for which she or he may not have the time or ability to assess based its scientific merits. As such, we assume that our experimental subjects are using partnership information as a heuristic cue to help make sense of the likely fairness of the partnership to which they are randomly assigned.

Although much fairness research centers on workplace settings, scholars also consider activities in legal (e.g., [45, 52, 53]) and political (e.g., [54, 55, 56]) contexts. Of particular relevance here is recent research that adapts the study of fairness to public perceptions of scientific topics involving perceived health and environmental risk [57–62].

### The current research

We perform three experimental studies to examine how different combinations of partners in a research collaboration influence subjects’ perceived procedural fairness and perceived legitimacy for that collaborative partnership. Although it is possible to imagine a range of ways to characterize a research partnership, we focus here on what we think is the most basic information about a partnership that might be communicated to create or alleviate concerns about conflict of interest: who is performing the research.

First, procedural justice scholarship and past research on conflict of interest suggests that a collaboration with industry or supported by industry funding will be seen as less fair and, through fairness, less legitimate. We formally state this as a two-part hypothesis describing fairness perceptions as a mediator.

Including an industry partner in a research collaboration (Hypothesis1a) directly reduces perceived fairness and (H1b) indirectly reduces perceived legitimacy.

It is less clear, however, how including other partners will influence fairness and legitimacy perceptions. As mentioned earlier, we consider three additional types of collaborators—university scientists, government agency scientists, and NGO scientists—on their own and in combination. Regarding the former, some recent research finds that university scientists generally have positive ratings of trust or fairness [63, 64]. Thus, it seems likely that including university scientists in a collaboration will have a positive effect on fairness and legitimacy perceptions.

Including a university partner in a research collaboration (H2a) directly increases perceived fairness and (H2b) indirectly increases perceived legitimacy.

Past research on conflict of interest does not, however, examine how people perceive government agency or NGO partners. The limited research on trust in scientists reveals that both of these groups are seen more positively than are industry scientists but less positively than are university scientists (e.g., [65, 66]). Given the lack of clear theoretical or empirical direction, we pose the effects of these partners as research questions.

What effect does including a government agency partner in a research collaboration have (Research Question 1a) directly on perceived fairness and (RQ1b) indirectly on perceived legitimacy?

What effect does including an NGO partner in a research collaboration have (RQ2a) directly on perceived fairness and (RQ2b) indirectly on perceived legitimacy?

## Study 1

### Methods

The Institutional Review Board of the Human Research Protection Program at Michigan State University approved this research (IRB#x15-167e)(exempt).

#### Stimulus

Study 1 subjects were told that we wanted to learn their views about “a potential new cooperative research partnership aimed at studying the possible negative health impacts of low levels of transfats in food.” “S1 Text Stimulus.” The only part of the message that varied across the experimental conditions was the specific combination of the following partners:

- Kellogg’s (a food company);
- Purdue University;
- The U.S. Centers for Disease Control and Prevention (CDC, a government agency); and
- The Union of Concerned Scientists (a non-governmental organization).

The 15 experimental conditions included each possible combination of one, two, three, and four of these partners. We selected these four organizations as representatives of agrifood businesses, research universities, governmental agencies, and NGOs, respectively, given the results of a pre-test with undergraduate students at a large Midwestern university “S1 Table.” Briefly, the pre-test participants viewed these four organizations more positively and less negatively than they viewed similar organizations in these four sectors.

#### Data collection

We administered our experiment via Qualtrics to subjects we recruited via Amazon Mechanical Turk (AMT), a crowdsourcing website where “requesters” solicit “workers” to perform “human intelligence tasks” (HITs) for pay. AMT has emerged as a practical way for recruiting a large number of participants from a reasonably wide cross-section of the general public—considerably more diverse than the traditional experiment recruitment pools of university undergraduates or surrounding community residents—either for online experiments (e.g., [67]) or for designing and testing new measurement instruments (e.g., [68]) across the social sciences (e.g., [69, 70]). Although the external validity of crowdsourced samples is limited (especially when compared to the generalizability of rigorously drawn representative samples), several studies confirm the value of AMT samples for assessing internal validity related to cause and effect, especially with between-subject experiments [70–72]. Further, we see no *a priori* reason to expect that the pattern of results found using our AMT sample would vary substantially with a different sample.

To solicit a broad cross-section of research subjects and minimize self-selection by AMT workers highly interested in food issues, we advertised a HIT titled “Industry Views Survey.” We limited participation to adults residing in the United States. Because this was an online experiment, the first page of the questionnaire included the approved consent statement, and subjects were asked to indicate consent by continuing with the questionnaire.

Subjects completed our experiment between April 19 and May 15, 2015. They earned $0.40 for participating, and the average response time was approximately seven minutes. We used two factual comprehension questions to screen out those subjects who failed to correctly identify the collaborative partners mentioned in their stimulus text. Our final sample includes 526 subjects who correctly answered both questions. An *a priori* power analysis for one-way ANOVA (main effects and interactions) with 15 groups conducted using G*Power [73], with a desired alpha of .05 and a power of .90 for a small/medium effect size (f = 0.15), shows that 469 respondents were needed for the current design. Approximately 53% of these subjects are female, 80% are white, and the average age is 39 (*SD* = 13). Also, about 27% of these subjects only have a high school diploma or GED, another 16% have earned up to an Associate’s degree, 43% hold a Bachelor’s degree, and an additional 4% have earned a doctorate or PhD.

#### Measures

As noted, we operationalized conflict of interest as *perceived procedural fairness* with a scale of seven items adapted from Leventhal [74]. Subjects indicated the extent to which they disagreed (“strongly disagree” = 1) or agreed (“strongly agree” = 7) that “the research partnership” will “ignore stakeholders that they disagree with” [reverse-coded], “listen to each other’s views,” “draw on the best available evidence,” “keep the best interests of consumers in mind,” “hide important findings if they don’t support their organizations” [reverse-coded], “work hard to avoid biasing their results,” and “slant their research to favor industry needs” [reverse-coded]. The order of these items was randomized. This perceived procedural fairness scale is highly reliable (Cronbach’s *alpha* = .93).

The perceived procedural fairness scale correlated highly (*r* = -.67, *p* <.01) with a single-item indicator of conflict of interest included in the experiment. For the latter, subjects reported whether they believed the partnership described would create “NO conflict of interest” = 1 to “a COMPLETE conflict of interest = 7 (*M* = 3.47, *SD* = 2.05). We used the perceived procedural fairness scale rather than this single-item indicator here because of the measurement advantages of multi-item scales and the conceptual desire to advance procedural fairness as a theoretically grounded way to study conflict of interest. The results of additional analyses using the single-item indicator (available upon request) are similar to what we present below.

We measured *perceived legitimacy* with a scale of three items asking subjects about how the proposed research should be used. Subjects indicated the extent to which they disagreed (“strongly disagree” = 1) or agreed (“strongly agree” = 7) with these three items: “I would make decisions about my diet based on the results of this partnership,” “government should pay attention to the results of this research partnership,” and “I would share the results of this research with people I know.” The order of these items was randomized. This perceived legitimacy scale is reliable (Cronbach’s *alpha* = .79).

The results of exploratory factor analysis using Maximum Likelihood Estimation and Varimax rotation (available upon request) indicate that the three perceived legitimacy items are distinct from the seven perceived procedural fairness items. Yet, the two scales are highly correlated (*r* = .64, *p*<0.01).

### Analyses

We conducted most of our analyses with SPSS 22. We first performed a one-way ANOVA with post-hoc tests looking only at main effects. The results of a General Linear Model (GLM) analysis (not shown) suggested no substantial interaction effects between the various conditions and we therefore focus here on main effects in the context of the hypothesized mediation. To this end, after looking at means, we conducted a more detailed analysis using the PROCESS macro for testing simple mediation [75]. PROCESS is well-established add-on to statistical packages such as SPSS that uses ordinary linear regression to estimate direct and indirect relationships and test for mediation. It can also be used to probe interactions. We ran this mediation model (with 1,000 bootstrap samples) four times to obtain estimates of both direct and indirect effects associated with having each type of organization involved in the partnership. Bootstrapping allows for the calculation of confidence intervals for indirect effects. These confidence intervals can be used to assess the statistical significance, something not possible in older approaches to assessing mediation such as those popularized by Baron and Kenny [76]. Mediation analyses such as these cannot confirm that fairness perceptions cause legitimacy perceptions, but the ordering is consistent with the underlying literature. Both variables are also treated as continuous variables for the purpose of linear modeling and, as the independent variables are dichotomous, the provided coefficients can be understood as the effect of including that partner in the partnership on the dependent variable. We include the PROCESS analyses as the primary assessment of the degree to which the results are at least consistent with our hypotheses and research questions.

Additional ways of analyzing the data (e.g., including a measure for the number of partners, or a measure for the number of non-industry partners in a regression model, not shown) similarly provided little additional explanatory power. Also, as noted above, we focus here on the separate main effect of each potential collaborator in the research partnership on both perceived procedural fairness and, ultimately, perceived legitimacy. Using Mplus 7, we also performed structural equation modeling (SEM) using latent variables for perceived procedural fairness and perceived legitimacy. These SEM results—which are displayed in “S2 Table”—are similar to PROCESS analyses.

### Results

Table 1 reports post hoc analyses associated with an initial one-way, between-subjects ANOVA for Study 1. The one way ANOVA test for main effects found that there were statistically significant but somewhat small differences in main effect between the various types of partnerships, both for perceived procedural fairness of the research process [*F*(511, 14) = 6.90, *p* < .01, η^2^ = .16)] and for perceived legitimacy of the resulting research [*F*(511, 14) = 4.17, *p* < .01, η^2^ = .10].

**Table 1:** Perceived procedural fairness and perceived legitimacy of partnership to investigate the health effects of small amounts of transfats in foods, ordered by mean of perceived procedural fairness with ANOVA post-hoc comparisons (N = 526).

	Perceived Procedural Fairness			Perceived Legitimacy		
	Mean	Standard Error	Sig. Diff.? (Tukey HSD)	Mean	Standard Error	Sig. Diff.? (Games-Howell)
Kellogg’s (n = 30)	3.64	0.28	a	3.31	0.26	a
Kellogg’s + CDC (n = 29)	4.17	0.29	ab	3.99	0.30	ab
Kellogg’s + UCS (n = 36)	4.18	0.20	ab	4.31	0.20	ab
Kellogg’s + Purdue + CDC (n = 44)	4.23	0.20	abc	4.44	0.23	ab
Kellogg’s + Purdue (n = 41)	4.23	0.23	abc	3.92	0.23	a
Kellogg’s + CDC + UCS (n = 33)	4.26	0.26	abcd	3.97	0.28	ab
Kellogg’s + Purdue + CDC + UCS (n = 26)	4.56	0.21	bcd	4.40	0.25	ab
Kellogg’s + Purdue + UCS (n = 26)	4.82	0.26	bcd	4.23	0.30	ab
Purdue + CDC (n = 38)	4.86	0.21	bcd	4.53	0.19	b
CDC (n = 32)	4.91	0.24	bcd	4.84	0.23	b
Purdue (n = 36)	5.17	0.17	bcd	4.56	0.15	b
Purdue + CDC + UCS (n = 35)	5.29	0.21	cd	5.02	0.21	b
UCS (n = 39)	5.42	0.18	d	4.79	0.27	b
CDC + UCS (n = 33)	5.44	0.19	d	5.29	0.20	b
Purdue + UCS (n = 38)	5.48	0.19	d	4.77	0.24	b

Notes: Shared letters indicate no significant difference (for example, two conditions that are both labeled ‘a’ are not significantly different. However, conditions that have an ‘a’ but no ‘b’ are significantly different from conditions that have a ‘b’ but no ‘a.’

Table 1 provides the results of post-hoc Tukey HSD tests for the perceived procedural fairness comparisons and the results of Games-Howell tests for the perceived legitimacy comparisons. We used two different tests to assess pairwise differences between the various conditions because Levene tests suggested equal variances between conditions for the perceived procedural fairness variable [*F*(511, 14) = .80, *p* = .67] but unequal variances for the perceived legitimacy variable [*F*(511, 14) = 1.94, *p* = .02]. The results of these tests indicate that including Kellogg’s in a research partnership investigating the health effects of small amounts of transfats in foods decreases both perceived procedural fairness and perceived legitimacy of the proposed research. Indeed, the partnerships with the eight lowest means for perceived procedural fairness and for perceived legitimacy each include Kellogg’s. The impacts of the inclusion of other partners are less clear in Table 1, though it does appear that partnerships including the UCS are perceived as fairer and more legitimate than partnerships that do not include the NGO.

Table 2 presents the results of the PROCESS mediation model for Study 1. Such models are based on Ordinary Least Squares regression but include two parts; the first part involves predicting the initial outcome that is hypothesized as the mediator (perceived procedural fairness, in this case) and the second part involves predicting the outcome that is the ultimate dependent variable (perceived legitimacy, in this case). In both cases, the estimates can be understood as unstandardized regression coefficients. The PROCESS macro then also provides a calculation of the overall direct relationship between the various independent variables and the indirect effect that is accounted for through the mediator with bootstrapping used to provide 95% confidence intervals in the absence of a statistical test for indirect effects. Statistical significance for the indirect effects, in this regard, can thus be understood as confidence intervals that do not include zero.

**Table 2:** Results of PROCESS mediation models for studies 1 and 2.

	Transfats in Food (Study 1) (N = 526)			New GM Food Grains (Study 2) (N = 627)		
	B	SE	Sig	B	SE	Sig
*Outcome*: *Perceived Procedural Fairness*						
(Constant)	**4.88**	**0.15**	**0.00**	**4.77**	**0.14**	**0.00**
Partnership Includes Kellogg’s	**-0.92**	**0.11**	**0.00**	**-0.73**	**0.11**	**0.00**
Partnership Includes Purdue	0.21	0.12	0.07	0.18	0.11	0.09
Partnership Includes CDC	-0.02	0.11	0.87	-0.03	0.11	0.77
Partnership Includes UCS	**0.41**	**0.12**	**0.00**	**0.27**	**0.11**	**0.01**
R^2^	0.15			0.09		
*Outcome*: *Perceived Legitimacy*						
(Constant)	**1.17**	**0.23**	**0.00**	**1.81**	**0.20**	**0.00**
Perceived Procedural Fairness	**0.66**	**0.04**	**0.00**	**0.60**	**0.03**	**0.00**
Partnership Includes Kellogg’s	-0.07	0.11	0.51	-0.09	0.09	0.34
Partnership Includes Purdue	-0.02	0.10	0.81	0.14	0.09	0.12
Partnership Includes CDC	**0.28**	**0.10**	**0.00**	0.12	0.09	0.17
Partnership Includes UCS	0.06	0.10	0.54	-0.01	0.09	0.90
R^2^	0.42			0.61		
*Summary of Direct and Indirect Effects*						
Direct Effects of Kellogg’s	-0.07	0.11	0.51	-0.09	0.09	0.34
Indirect Effects of Kellogg’s	**-0.61**	**0.08**	**[-0.79, -0.46]**	**-0.44**	**0.07**	**[-0.61, -0.32]**
Direct Effects of Purdue	-0.02	0.10	0.81	0.14	0.09	0.12
Indirect Effects of Purdue	0.14	0.07	[-0.01, 0.30]	0.11	0.07	[-0.03, 0.24]
Direct Effects of CDC	**0.28**	**0.10**	**0.00**	0.12	0.09	0.17
Indirect Effects of CDC	-0.01	0.08	[-0.16, 0.14]	-0.02	0.06	[-0.15, 0.11]
Direct Effects of UCS	0.06	0.10	0.54	-0.02	0.09	0.90
Indirect Effects of UCS	**0.27**	**0.07**	**[0.12, 0.41]**	**0.16**	**0.07**	**[0.03, 0.30]**

Notes: Confidence ranges for indirect effects are based on bootstrapping (n = 1,000) with significance interpreted as situations in which the range does not include 0.00.

Overall the Study 1 model explains 15% of the variation in perceived procedural fairness and 42% of the variation in perceived legitimacy. These results, more than the mean comparisons, clearly convey the effect of including Kellogg’s in a research partnership investigating the health effects of small amounts of transfats in foods. Including Kellogg’s leads to an almost 1-unit decrease in the 7-unit scale for perceived procedural fairness (supporting H1a). The test of indirect effects in the bottom part of Table 2 shows that the inclusion of Kellogg’s has a statistically significant negative indirect effect on perceived legitimacy through its negative effect on perceived procedural fairness (supporting H1b).

Further, including the UCS in a partnership leads to a 0.41-unit increase in perceived procedural fairness (RQ2a). This inclusion also has a positive effect on perceived legitimacy via the indirect, positive path through perceived procedural fairness (RQ2b). Including Purdue University in a partnership has no statistically significant effect on either perceived procedural fairness or perceived legitimacy, offering no support to H2a and H2b, respectively. Also, including the CDC in a partnership has no statistically significant effect on perceived procedural fairness (RQ1a), but it does have a positive, direct effect on perceived legitimacy (RQ1b).

Overall, Study 1 clearly supports H1a and H1b. Including a private sector representative in a research partnership decreases the extent to which people perceive the collaboration as procedurally fair. Further, the pattern of results is consistent with the idea that this negative impact may reduce the extent to which people perceive the research results as a legitimate source of knowledge for use in their lives. Of course, these results may somewhat be an artifact of the particular issue we examined in Study 1. To allay such concern, we conducted a second, nearly identical experiment that focused on a different health science issue.

## Study 2

For Study 2, we chose to focus on the health impacts of genetically modified (GM) food. Industry funding of GM food research has received considerable public attention (e.g., [6]). Further, science and risk communication scholars [77] are devoting more attention to the topic, even applying procedural justice scholarship to understand public perceptions [57]. To aim for wider generalizability across Studies 1 and 2, we further clarified that the proposed research on GM food would investigate possible positive health impacts of new GM grains (e.g., rice, wheat, corn) that are being designed to absorb less toxic substances from the soil than do current grains. “S2 Text Stimulus.” This focus on the potential positive health impacts of GM food in Study 2 may tap different dynamics than does the focus on the potential negative health impacts of small amounts of transfats in food in Study 1. (The consent process was identical to Study 1).

### Methods

As with study 1, the Institutional Review Board of the Human Research Protection Program at Michigan State University approved this research (IRB#x15-167e)(exempt).

Other than shifting the substantive focus from transfats to GM food and replacing the CDC with the U.S. Food and Drug Administration (FDA) as the governmental agency partner, Study 2 employed the same design and identical measures to those used in Study 1. We again administered our experiment via Qualtrics to adult U.S. residents we recruited via AMT with a HIT titled “Collaboration between Different Research Partners.” Subjects completed our experiment between July 8 and July 14, 2015, and earned $0.75 for participating. The average response time was approximately eleven minutes. After excluding subjects who participated in previous studies, our final sample includes 627 subjects who correctly identified the collaborative partners mentioned in their stimulus text. For a discussion of power, see Study 1. Approximately 55% of subjects are female, 88% are white, and the average age is 37 (*SD* = 13). Also, about 31% of subjects have only a high school diploma or GED, another 14% have earned up to an Associate’s degree, 42% hold a Bachelor’s degree, and an additional 3% have earned a doctorate or PhD.

We measured conflict of interest as *perceived procedural fairness* with the same 7-item scale (*Cronbach’s alpha =* .*93)* and perceived legitimacy with the same 3-item scale *(Cronbach’s alpha =* .*81)* that we used in Study 1. As in Study 1, the results of exploratory factor analysis using Maximum Likelihood Estimation and Varimax rotation (available upon request) indicate that the three perceived legitimacy items are distinct from the seven procedural fairness items. Yet, the two scales are highly correlated (*r* = .61, *p* < .01). Also, the correlation between a 7-point single-item indicator of conflict of interest and our perceived procedural fairness scale was again quite high (r = -.60, p < .01). We also performed SEM using latent variables for perceived procedural fairness and perceived legitimacy. These SEM results, which are displayed in S2 Table, are similar to those presented in Table 2.

### Results

Table 3 again reports means and post-hoc test results from a one-way, between-subjects ANOVA results for Study 2. As with Study 1, the omnibus results reveal statistically significant main effect differences between the various types of partnerships both for perceived procedural fairness of the research process [*F*(612, 14) = 5.10, *p* < .01, η^2^ = .10)] and for perceived legitimacy of the resulting research [*F*(612, 14) = 3.80, *p* < .01, η^2^ = .08)]. This pattern of results, coupled with the post-hoc tests also reported in Table 3, indicates that including Kellogg’s in a research partnership investigating the health impacts of new GM grains decreases both the perceived procedural fairness (H1a) and perceived legitimacy (H1b) of the proposed research.

**Table 3:** Perceived procedural fairness and perceived legitimacy of partnership to investigate the health effects of new genetically modified food grains, ordered by mean of perceived procedural fairness with ANOVA post-hoc comparisons (N = 627).

	Perceived Procedural Fairness			Perceived Legitimacy		
	Mean	Standard Error	Sig. Diff.? (Tukey HSD)	Mean	Standard Error	Sig. Diff.? (Games Howell)
Kellogg’s + Purdue + CDC (n = 48)	3.95	0.22	a	4.21	0.23	ab
Kellogg’s (n = 37)	3.95	0.20	a	4.47	0.21	abc
Kellogg’s + UCS (n = 34)	4.01	0.24	ab	3.75	0.26	a
Kellogg’s + CDC (n = 42)	4.14	0.22	abc	4.33	0.24	abc
Kellogg’s + Purdue (n = 32)	4.20	0.22	abc	4.33	0.19	abc
Kellogg’s + CDC + UCS (n = 44)	4.43	0.20	abcd	4.48	0.25	abc
Kellogg’s + Purdue + UCS (n = 44)	4.58	0.20	abcd	4.77	0.19	abc
CDC + UCS (n = 43)	4.77	0.20	abcd	4.84	0.20	bc
Kellogg’s + Purdue + CDC + UCS (n = 35)	4.80	0.21	abcd	4.98	0.25	bc
Purdue + CDC (n = 46)	4.86	0.20	abcd	4.77	0.19	abc
CDC (n = 43)	4.97	0.18	bcd	5.14	0.17	bc
Purdue + CDC + UCS (n = 44)	5.03	0.23	cd	5.23	0.22	bc
Purdue (n = 42)	5.05	0.22	cd	4.98	0.20	bc
UCS (n = 43)	5.12	0.18	cd	4.66	0.21	abc
Purdue + UCS (n = 50)	5.32	0.16	d	5.25	0.17	c

Notes: Shared letters indicate no significant difference (for example, two conditions that are both labeled ‘a’ are not significantly different. However, conditions that have an ‘a’ but no ‘b’ are significantly different from conditions that have a ‘b’ but no ‘a.’

Table 2 also presents the results of the PROCESS mediation model for Study 2. Overall this model explains 9% of the variation in perceived procedural fairness and 61% of the variation in perceived legitimacy. These results of the PROCESS mediation model clearly convey the effect of including Kellogg’s in a research partnership investigating GM grains. Including Kellogg’s leads to a 0.73-unit decrease in perceived procedural fairness (supporting H1a). The test of indirect effects in the bottom part of Table 2 suggests that the inclusion of Kellogg’s has a statistically significant negative indirect effect on perceived legitimacy through its negative effect on perceived procedural fairness (supporting H1b).

As in Study 1, including the UCS in a partnership leads to an increase in perceived procedural fairness (RQ2a). The results are again consistent with a potential positive effect of this partner on perceived legitimacy via the indirect, positive path through perceived procedural fairness (RQ2b). Including Purdue in a partnership has no statistically significant effect on either perceived procedural fairness or perceived legitimacy, offering no support to H2a and H2b, respectively. While including a governmental agency (the CDC) in a partnership has a positive, direct effect on perceived legitimacy (RQ1b) in Study 1, it does not have such an effect in Study 2 (the FDA).

The results of Study 2 largely confirm those of Study 1, increasing our confidence in the patterns we have found. Given the size and consistency of this effect, we conducted a third experiment to gather some qualitative data to better understand *why* subjects perceive the inclusion of a private sector partner with such skepticism.

## Study 3

### Methods

As with studies 1 and 2, the Institutional Review Board of the Human Research Protection Program at Michigan State University approved this research (IRB#x15-167e)(exempt).

Study 3 used the same transfats stimulus and between-subjects design with 15 randomly assigned conditions as in Study 1. After providing consent in the same manner as in Studies 1 and 2, subjects read their assigned description of a proposed partnership to examine the effects of small amounts of transfats in foods and answered the same three comprehension questions as used in Study 1. The remainder of the experiment simply asked subjects to respond to three open-ended questions about the partnership and its research. Below, we describe the results of a content analysis of responses to the following question.

Please share your views about the nature of the proposed research partnership itself. In your answer, please address the following. What are the strengths and limitations of the proposed partnership? Please fully explain your answer, including any positive or negative thoughts you have about any specific partners.

The other two questions addressed perceived impacts of the research and the types of activities the respondent thought might help mitigate potential problems and were meant to assist with future research. These are not addressed here.

We administered our experiment via Qualtrics to adult U.S. residents we recruited via AMT with a HIT titled “Answer a Few Questions about a Proposed Scientific Partnership.” Our experiment was completed by 305 U.S. residents on May 31, 2015. Subjects earned $0.75 for completing the experiment, which took slightly less than eight minutes on average. After excluding subjects who participated in Study 1, our final sample includes 222 subjects who provided correct answers to three comprehension questions. That is, they (a) knew that food producers use transfats to give food softer texture, (b) knew that the described study would examine if small amounts of transfats are unhealthy, and (c) correctly identified the presence or absence of Kellogg’s in the proposed partnership.

A co-author well-experienced in qualitative research performed the qualitative data analysis. Given that our specific objective (to better understand why inclusion of a private sector partner engendered heightened skepticism) was fairly straightforward and the data structure (brief responses to a single open-ended question) was relatively simple, we decided that multiple coders were unnecessary. Our co-author employed an inductive coding scheme to analyze responses to the italicized question above. First, he read all the responses, coding them for the presence or absence of negative, positive, and/or neutral comments. In doing so, he also identified 13 substantive themes that emerged from subjects’ responses [78]. With this list of potential themes, he then re-read all the responses and coded them for the presence or absence of each substantive theme. Approximately 11% of responses contained no theme, nearly 49% contained just one, about 35% contained only two, and approximately 6% contained three themes. Below, we report and discuss those seven substantive themes appearing in at least 10% of responses.

### Results

Given our interest in public reaction to the inclusion of a private sector representative in a scientific partnership, we analyzed subjects’ responses in two subsamples: subjects who responded to partnerships including Kellogg’s (120 or 54%) and those who responded to partnerships that did not include Kellogg’s (102 or 46%). When the proposed partnership included Kellogg’s, 77% (92 of 120) of responses included a negative comment, 54% (65) included a positive comment, and 4% (5) remained neutral with neither a clearly positive nor clearly negative comment. When the proposed partnership did not include Kellogg’s, only 28% (29) of responses included a negative comment, 79% (81) of responses included a positive comment, and 9% (9) of responses remained neutral. The rather large difference in the percentage of negative responses across the two subsamples (48%) generally confirms the patterns revealed in Studies 1 and 2.

Table 4 reports the most prevalent themes—with italicized illustrative examples—about the proposed partnership that emerged from our content analysis. The percentages in Table 4 sum to more than 100, since subjects’ responses may contain more than one theme.

**Table 4:** Most prevalent themes (and illustrative examples) about the proposed partnership to study the negative health impacts of low levels of transfats in food.

When the Partnership includes Kellogg’s (N = 120)	% Mentioning
Kellogg’s Is a Problematic Partner [I]t seems that [Kellogg’s] may have an incentive to skew results to maximize their profits. [Kellogg’s has] a vested interest in the outcome so the results would be completely invalid in my eyes.… just the fact that a food company is involved makes me think of the tobacco industry and all that corruption.	69
The Non-Kellogg’s Partners May Provide At Least Some Checks on Kellogg’s Inclusion of a food company that has a vested financial interest in the result is a pretty big limitation on the validity of the results. On the other hand, [UCS] and CDC might provide a reasonable check and balance…Kellogg’s… has a vested interest in being able to continue to use transfats. The other 3 organizations involved do not have the same financial incentive and thus will hopefully help balance out this bias.	30
The Partnership Is At Least Somewhat Good I believe this would be a good partnership. This would provide different sides to the research so an unbiased result can be made. Overall it looks like a strong partnership.	27
**When the Partnership Does Not Include Kellogg’s (N = 102)**	% Mentioning
The Partnership Is At Least Somewhat Good The partnership includes a wide variety of interests. I believe that conflicts of interest can be avoided because of this. It sounds like a good unbiased partnership. The 3 partners have nothing to gain from the results of the study and 1 is a government group.	67
The Partnership Has Little or No Conflict of Interest or Bias I see no conflict of interest or any reason to believe (at this time) that their study would be conducted in any way other than in an honest and unbiased manner. The strengths would be that fair research would be done without conflict of interest.	25
Concern about Potential or Future Conflict of Interest or Bias There is always a chance that there may be a (monetary) conflict of interest with some of the scientist involved. Unfortunately money is the root of all BAD studies. While I like the partnership between academia and the federal government I am still concerned that there may or may not be an “agenda” or pre-conceived results desired from this study.	17
The Partners’ Differences Will Balance Out I don’t think any institution or government agency or scientist can be trusted anymore to make any claims without political interest. Maybe by combining them, there can be some balance. The strengths would be that scientists from different organizations would be coming together, which would hopefully expand expertise and decrease any conflicts of interest.	12

Note: Percentages sum to more than 100, since subjects may mention more than one theme in their responses.

#### With Kellogg’s as a partner

Three major themes emerged from responses by subjects exposed to partnerships including Kellogg’s. Almost 70% of these subjects noted that Kellogg’s is so problematic as a partner that its mere inclusion may call the entire partnership into question. The presence of Kellogg’s in the partnership provoked distrust and skepticism among many subjects. Indeed, when these subjects mentioned both positive and negative aspects of the partnership, the positives nearly always referred to partners or features other than Kellogg’s, and the negatives nearly always referred to Kellogg’s. Some assumed that Kellogg’s would likely try to skew the research results to their private interests and/or conceal undesirable results. Kellogg’s was described as “clearly,” “very,” “extremely,” “incredibly,” and “strongly” biased with an “ulterior motive,” “profit motive,” "vested financial interest,” and “conflict of interest.”

Almost one-third of responses (30%) claimed that other groups in the partnership might provide at least some checks on the financial influence and possibly dubious activities of Kellogg’s. These subjects believed that non-Kellogg’s partners would add scientific credibility and honesty to the research, while reducing the likelihood that Kellogg’s bias would influence methods and results. Subjects sometimes expected, and at other times merely hoped, that this would be the case. Generally, subjects seemed more confident in Purdue’s ability to counterbalance Kellogg’s influence than in the abilities of the CDC or UCS to do so. Indeed, a few subjects even acknowledged that other partners (notably, the CDC) may be easily manipulated by Kellogg’s.

Finally, about one-fourth of subjects (27%) mentioned that the partnership was at least minimally good, even if giving reservations. These responses described the partnership as “good,” “great,” “worthwhile,” “strong,” and “balanced.” They noted that the diverse membership of organizations working together would bring different interests and points of view together to strengthen the resulting research.

#### Without Kellogg’s as a partner

Four major themes emerged from the responses of subjects exposed to partnerships not including Kellogg’s. The most prevalent theme, found in 67% of responses, was that the partnership was at least somewhat good—as illustrated by responses calling the partnership “a very good idea,” “fruitful,” “very beneficial,” “greatly beneficial,” and “great.” Several subjects voiced the adage that “more heads are better than one,” noting that multiple partners assure thoroughness and that partnerships can often achieve greater advances than when just one actor goes it alone. Overall, a good number of responses conveyed the expectation that the partnership would produce important, reliable, and useful results that would improve our understanding of the health risks of transfats.

Approximately one-fourth of subjects (25%) explicitly noted that the partners seemed immune to outside influence and had little or no conflict of interest or bias. Subjects claimed that the partners seem “fairly objective,” “like relatively neutral parties,” and “an unbiased group” with no clear agenda. Key here seemed to be the perception of no obvious interests in the outcome of the research, since the partners were not funded or employed by food companies. Because of this, subjects expected that the research would be performed in “an honest and unbiased manner.”

On the other hand, a third theme (found in 17% of responses) expressed concern about potential conflict of interest or bias. Subjects conveyed either general concerns about the broad influence of money or the potential for one partner to dominate another, or they focused more specifically on the potential financial conflicts of interests of individual scientists. Yet, a final theme (found in 12% of responses) claimed that the differences between the partners would balance out and their different points of view would likely neutralize any potential conflicts of interest or bias. Such subjects argued that one or more partners would serve as a counterbalance to any pressure another partner would potentially assert to bias the methods or results.

## Conclusion

Private industry now dominates U.S. R&D funding [1], and university scientists are under increasing pressure to seek non-government funding and participate in public-private partnerships with private sector representatives [2–4]. Yet, much research has documented that in some cases industry-funded research may be less than credible, especially where the risk of regulation is nontrivial (e.g., [8, 9, 11, 79]). Further, public skepticism of industry-funded research—heightened by revelations about bias in such work—may likely constrain the efficacy and influence of its results (e.g., [5, 6]).

We performed three experiments to investigate public perception of industry-funded research. Building upon efforts to adapt procedural justice scholarship to the study of health and risk communication (e.g., [57, 59, 80]), we drew insights from procedural justice research to examine how the inclusion of an industry partner in a scientific collaboration influences the perceived procedural fairness of the partnership and the perceived legitimacy of the resulting research.

We found fairly consistent results across Studies 1 and 2. In both experiments, including Kellogg’s in a scientific partnership indirectly decreased perceived legitimacy of the partnership’s research via its negative effect on the perceived procedural fairness of the partnership. Also in both experiments, including an NGO (UCS) in a scientific partnership indirectly increased perceived legitimacy via its positive effect on perceived procedural fairness. Neither the inclusion of Purdue University nor the inclusion of a government agency (the CDC or the FDA) in the scientific partnership had a consistent effect on perceived procedural fairness or perceived legitimacy across both studies.

The results of Study 3 provided qualitative insights for better understanding the negative effect of including Kellogg’s on both the perceived procedural fairness and perceived legitimacy of the scientific partnership. Subjects exposed to a scientific partnership including Kellogg’s were nearly three times more likely to offer a negative comment about the partnership than were subjects exposed to a scientific partnership not including Kellogg’s. The former group of subjects expressed deep concern that Kellogg’s financial interests would lead it to skew the results of the partnership in ways favorable for the company. Although some subjects hoped that including other actors—whether research universities, government agencies, or NGOs—in the partnership might reduce this potential for bias, few reported with confidence that this would actually happen. In contrast, when Kellogg’s was not included in the partnership, subjects generally believed that the resulting research would be conducted fairly.

We end by briefly outlining an agenda for moving this scholarship forward. First, additional research should aim to replicate this study to more completely assess the consistency and generalizability of our results. Such future research could (a) explore a wider array of substantive areas (e.g., environmental impacts associated with new technologies, health impacts of industrial chemicals, effectiveness of drugs or supplements), (b) consider different kinds of potential research partners, and (c) examine whether it matters what the partnership ultimately finds. Procedural justice scholarship could provide additional insight here inasmuch as past results suggest that people may discount results they disagree with if they learn about these results before learning about the processes that lead to the results [47]. Another important avenue of research would (d) involve considering additional procedures (e.g., independent advisory boards, transparency tools, etc.), beyond partnerships, that organizations might use either on their own or in combination. Also, as noted above, it may be worth considering additional work related to the question of interactions between partners or the effects of adding multiple partners. As noted, we conducted initial analyses on these types of questions using the data here, but ultimately we decided to focus on the main effects of each partner. We saw little evidence that the combinations mattered, and it therefore made more sense to us to focus on the most parsimonious model for this initial study. However, this should not be taken as strong evidence that it does not make sense to consider partnership combinations both for research and in real life.

Second, given the sizable contribution of industry funding to scientific research in the U.S. and especially if additional studies replicate our results, future multidisciplinary work should investigate historical and contemporary efforts to implement procedures that aim to reduce conflicts of interest due to industry involvement. This may include both historical and sociological research into what organizations have done or are doing, as well as potentially identifying the additional procedures (or combinations of procedures) that might be tried. Insights from procedural justice scholarship may further guide this research. For example, those interested in the cognitive mechanisms behind fairness perceptions could test, for example, whether communicating about conflict of interest mitigation procedures are more effective when subjects are forced to think heuristically or when they are more uncertain about the underlying topics (e.g., [81]).

## Supporting information

S1 Table Descriptive statistics from the pre-test of potential research partners. (DOCX)

S2 Table Unstandardized direct, indirect, and total effects from structural equation model predicting perceived legitimacy (latent variable) by presence of collaborative partner with perceived procedural fairness (latent variable) as mediator. (DOCX)

S1 Text Stimulus. Study 1 stimulus with manipulated text italicized.
(DOCX)

S2 Text Stimulus. Study 2 stimulus with manipulated text italicized.
(DOCX)
