# Correction: Perceived conflict of interest in health science partnerships

**DOI:** 10.1371/journal.pone.0202392

**Published:** 2018-08-10

**Authors:** The PLOS ONE Staff

The Data Availability statement is incorrect. The correct statement is: Data are available at https://doi.org/10.3886/ICPSR37033.v1. The publisher apologizes for the error.
