# Supplementary material for: Perceived conflict of interest in health science partnerships
Source: PLoS One. 2017 Apr 20;12(4):e0175643. doi: 10.1371/journal.pone.0175643 (PMC5398532; doi:10.1371/journal.pone.0175643)
Supplement: S1 Table — (DOCX) [file pone.0175643.s001.docx]

S1Table: Descriptive Statistics from the Pre-Test of Potential Research Partners

|  | **Positive Opinion** | | **Negative Opinion** | |
| --- | --- | --- | --- | --- |
|  | **Mean** | **Standard Error** | **Mean** | **Standard Error** |
| Agrifood Businesses |  |  |  |  |
| Kellogg’s | **3.66** | **0.09** | **2.30** | **0.11** |
| General Mills | 3.52 | 0.09 | 2.33 | 0.10 |
| Nestle | 3.50 | 0.10 | 2.55 | 0.11 |
| Kraft | 3.46 | 0.12 | 2.39 | 0.11 |
| ConAgra | 2.85 | 0.08 | 3.02 | 0.09 |
|  |  |  |  |  |
| Research Universities |  |  |  |  |
| Purdue University | **3.58** | **0.07** | **2.30** | **0.07** |
| Michigan State University | 3.57 | 0.08 | 2.33 | 0.07 |
| Colorado State University | 3.54 | 0.06 | 2.39 | 0.07 |
| University of Minnesota | 3.50 | 0.06 | 2.39 | 0.07 |
| Iowa State University | 3.46 | 0.06 | 2.42 | 0.07 |
|  |  |  |  |  |
| Governmental Agencies |  |  |  |  |
| U.S. Centers for Disease Control and Prevention | **3.83** | **0.09** | **2.25** | **0.10** |
| U.S. Environmental Protection Agency | 3.49 | 0.11 | 2.48 | 0.11 |
| U.S. Department of Agriculture | 3.33 | 0.10 | 2.72 | 0.10 |
| U.S. Food and Drug Administration | 3.30 | 0.11 | 2.83 | 0.11 |
|  |  |  |  |  |
| Non-Governmental Organizations |  |  |  |  |
| Union of Concerned Scientists | **3.47** | **0.07** | **2.50** | **0.08** |
| Center for Food Safety | 3.41 | 0.07 | 2.63 | 0.07 |
| Natural Resources Defense Council | 3.36 | 0.08 | 2.59 | 0.07 |
| Center for Science in the Public Interest | 3.31 | 0.07 | 2.63 | 0.07 |
|  |  |  |  |  |

Notes: Participants were asked whether they have a positive or negative opinion of each organization using a 5-point scale (“strongly disagree” = 1 to “strongly agree” = 5): “I have a positive opinion of _____” and “I have a negative opinion of _____”. Participants received the two questions and the items within each question in random order. N = 120-123.
