# Supplementary material for: Perceived conflict of interest in health science partnerships
Source: PLoS One. 2017 Apr 20;12(4):e0175643. doi: 10.1371/journal.pone.0175643 (PMC5398532; doi:10.1371/journal.pone.0175643)
Supplement: S2 Table — (DOCX) [file pone.0175643.s002.docx]

S2 Table: Unstandardized Direct, Indirect, and Total Effects from Structural Equation Model Predicting Perceived Legitimacy (Latent Variable) by Presence of Collaborative Partner with Perceived Procedural Fairness (Latent Variable) as Mediator

|  | **Perceived Legitimacy of Transfats Research (Study 1)** | | |  | **Perceived Legitimacy of GM Food Research (Study 2)** | | |
| --- | --- | --- | --- | --- | --- | --- | --- |
|  | **Direct** | **Indirect** | **Total** |  | **Direct** | **Indirect** | **Total** |
| Partnership Includes Kellogg’s | -0.01 (0.11) | -0.70* (0.10) | -0.71* (0.13) |  | -0.06 (0.10) | -0.50* (0.08) | -0.56* (0.12) |
| Partnership Includes Purdue | -0.03 (0.11) | 0.15* (0.09) | 0.12 (0.13) |  | 0.10 (0.10) | 0.14* (0.07) | 0.25* (0.12) |
| Partnership Includes CDC | 0.30* (0.10) | -0.02 (0.09) | 0.28* (0.13) |  | 0.12 (0.10) | -0.02 (0.07) | 0.10 (0.12) |
| Partnership Includes UCS | 0.03 (0.11) | 0.31* (0.09) | 0.34* (0.13) |  | -0.03 (0.10) | 0.20* (0.07) | 0.17 (0.12) |
|  |  |  |  |  |  |  |  |
| Perceived Procedural Fairness | 0.80* (0.06) |  | 0.80* (0.06) |  | 0.70* (0.06) |  | 0.70* (0.06) |
|  |  |  |  |  |  |  |  |
| Chi-Square/Degrees of Freedom |  |  | 168.87*/54 |  |  |  | 240.78*/54 |
| Root Mean Square Error of Approximation |  |  | 0.06 |  |  |  | 0.07 |
| Comparative Fit Index |  |  | 0.97 |  |  |  | 0.95 |
| Tucker-Lewis Index |  |  | 0.95 |  |  |  | 0.94 |
|  |  |  |  |  |  |  |  |
| R^2^ for Perceived Procedural Fairness |  |  | 0.56 |  |  |  | 0.49 |
| R^2^ for Perceived Legitimacy |  |  | 0.16 |  |  |  | 0.10 |
|  |  |  |  |  |  |  |  |
| Sample Size |  |  | 526 |  |  |  | 627 |

Notes: * *p* < .05 (one-tailed)
