# Supplementary material for: Perceived conflict of interest in health science partnerships
Source: PLoS One. 2017 Apr 20;12(4):e0175643. doi: 10.1371/journal.pone.0175643 (PMC5398532; doi:10.1371/journal.pone.0175643)
Supplement: S1 Text — Study 1 stimulus with manipulated text italicized. (DOCX) [file pone.0175643.s003.docx]

S1Text Stimulus: Study 1 Stimulus with Manipulated Text Italicized

Our goal is to understand your views about a potential new cooperative research partnership aimed at studying the possible negative health impacts of low levels of transfats in food.

This research will be funded and conducted by groups of researchers from:

- *Kellogg’s (a food company)*
- *Purdue University (a public research university)*
- *The U.S. Centers for Disease Control and Prevention (CDC, a government agency)*
- *The Union of Concerned Scientists (a non-governmental organization)*

*[Each subject received a combination of between one and four of these partners for a total of 15 conditions.]*

Recent research has shown that large quantities of transfats are unhealthy. However, little is known about the effects of eating small amounts of transfats. Those involved in food production sometimes use small amounts of transfats to give food a softer texture. Some groups have raised health concerns about even these small amounts of transfats.

This research would help decide whether transfats should be used. It could mean that some foods would not be sold or would cost more because of the need to use different ingredients.

Critics have also complained that past research results related to transfats may not be correct because of some researchers’ conflicts of interest.
