# Supplementary material for: Perceived conflict of interest in health science partnerships
Source: PLoS One. 2017 Apr 20;12(4):e0175643. doi: 10.1371/journal.pone.0175643 (PMC5398532; doi:10.1371/journal.pone.0175643)
Supplement: S2 Text — Study 2 stimulus with manipulated text italicized. (DOCX) [file pone.0175643.s004.docx]

S2 Text Stimulus: Study 2 Stimulus with Manipulated Text Italicized

Our goal is to understand your views about a potential new cooperative research partnership aimed at studying the possible health impacts of new genetically modified (GM) grains (e.g., rice, wheat, corn).

This research will be funded and conducted by groups of researchers from:

- *Kellogg’s (a food company)*
- *Purdue University (a public research university)*
- *The U.S. Food and Drug Administration (FDA, a government agency)*
- *The Union of Concerned Scientists (a non-governmental organization)*

*[Each subject received a combination of between one and four of these partners for a total of 15 conditions.]*

Research has shown that GM foods—sometimes call GMOs or genetically engineered crops—currently on the market are safe to eat. However, little is known about the health effects of the new GM grains that are being developed. They are being modified, however, so that they will be less likely than current grains to absorb toxic substances from the soil. This could make the GM grains healthier than conventional crops, but some groups have raised health concerns about GM crops.

This research would help decide whether the new GM grains should be used in food products. If the research identifies negative health impacts, it could mean that some foods would not be sold or would cost more because of the need to use different crops.

Critics have also complained that past research results related to GM foods may not be correct because of some researchers' conflicts of interest.
